# Support System for the Assessment and Intervention During the Manual Material Handling Training at the Workplace: Contributions From the Systematic Observation

**DOI:** 10.3389/fpsyg.2019.01247

**Published:** 2019-06-05

**Authors:** Mariona Portell, Anna M. Sene-Mir, M. Teresa Anguera, Gudberg K. Jonsson, José L. Losada

**Affiliations:** ^1^Department of Psychobiology and Methodology of Health Sciences, Universitat Autònoma de Barcelona, Cerdanyola del Vallès, Spain; ^2^Physical Activity and Sports Studies Centre, University of Vic – Central University of Catalonia, Vic, Spain; ^3^Faculty of Psychology, Institute of Neurosciences, University of Barcelona, Barcelona, Spain; ^4^Human Behavior Laboratory, University of Iceland, Reykjavik, Iceland; ^5^Faculty of Psychology, University of Barcelona, Barcelona, Spain

**Keywords:** systematic observation, health promotion in the workplace, T-pattern analysis, polar coordinate analysis, mixed method approach, manual material handling, feedback

## Abstract

Efficacy of classical manual material handling (MMH) training interventions on back pain prevention at the workplace has been called into question. The way that observation (self-observation or hetero-observation) is used in other areas to create feedback addressed to modify motor activities can justify innovative components for these interventions. However, their implementation and evaluation cannot be done without tackling the methodological challenge of developing a reliable observational instrument to measure manual handling practice during the training process. The aims of this study were: (1) justify and develop an hetero-observation (H-O) instrument to assess changes in the worker behavioral patterns with a level of analysis convenient to derive a parallel version for the systematic self-observation (S-O) during training on MMH; (2) provide evidence on the inter-rater reliability of the H-O instrument; (3) provide evidence on the usability of the S-O instrument and its perceived usefulness; and (4) provide evidence on the benefits that can be derived with the use of the H-O instrument to create feedback based on T-pattern and polar coordinate analysis. A mixed method approach mainly grounded on systematic observation was used. A convenience sample composed by blue-collar workers participated in the study. Based on literature review and expert opinion, the H-O instrument proposed was composed by six dimensions (feet, knee joints, back, elbow joints, load position, and interaction between back tilt and displacement) plus a structural dimension which defined MMH phases. The inter-rater reliability of this instrument was almost perfect for all dimensions using a tolerance level of 2 s (the range of time-unit kappa was from 0.93 to 0.97 and the range of event-based kappa was from 0.82 to 0.9). The usability and usefulness of the S-O instrument was highly valued by workers. Regarding the way to use hetero-observations to create feedback, the paper shows the great potential of T-pattern and polar coordinate analysis. The observational instruments developed combined with these techniques make it possible to characterize the body positions adopted during manual handling performance, and this is crucial to create feedback on performance instead of only feedback on results.

## Introduction

Musculoskeletal disorders (MSDs) are one of the most common occupational disorders worldwide (Schneider and Irastorza, 2010; Hossain et al., 2018; Penkala et al., 2018). Lifting and handling of loads have been associated with an increased risk of back disorders, mainly developing low back pain (Bernard, 1997; Hoogendoorn et al., 2000; Thiese et al., 2014). Even though the majority of the tasks have been automated, manual material handling (MMH) is still being carried out in numerous workplaces (e.g., building sites, nursing, or the food industry), and what is more, there are plenty of daily life activities in which people may perform MMH during non-working hours (e.g., lifting or carrying people or objects such as boxes).

Throughout the last decades, there has been an increase of studies focused on developing, implementing and evaluating the efficacy of MMH training on the reduction of low back pain or back injury prevention (Punnett et al., 2009; van der Beek et al., 2017). The systematic reviews of Clemes et al. (2010), Verbeeck et al. (2011), and Hogan et al. (2014) agreed that most of MMH trainings are not effective to reduce low back pain or back injury. This lack of effectiveness has been related to the focus on a task-specific training instead of a multidimensional approach (Clemes et al., 2010). Also, it has been related to the use of interventions not based on a behavioral change theory (Verbeeck et al., 2011; Hogan et al., 2014). Most studies have focused on evaluating the effectiveness of training on long-term results, such as reduction of MSDs, and there are few rigorous evaluations of the effect of MMH training on key intermediate variables of the changing health behavior theory, such as knowledge or behavior change (Hogan et al., 2014). The inclusion of these intermediate variables is necessary to evaluate the implementation process, which in turn is necessary to identify why an intervention worked or not, and under which operating conditions these interventions are likely to be most effective (Pedersen et al., 2012). Another limitation is the lack of transferability of the intervention effectiveness from the training situation to other labor and non-labor settings; and this lack of transferability has been related to the components of those interventions (Ford et al., 1998; Clemes et al., 2010; Hogan et al., 2014). Solving some of the aforementioned constraints cannot be done without tackling a methodological challenge related to the development of reliable observational instruments to measure manual handling practice during and after training (Hogan et al., 2014).

On the transferability issue, the way that observation (self-observation or hetero-observation) is used in other areas to create feedback addressed to modify motor activities can justify innovative approaches (Kernodle and Carlton, 1992; Janelle et al., 1997; Dowrick, 1999, 2012; Wulf et al., 2010; Magill, 2011; Ste-Marie et al., 2013). Previous research provided evidence on the effectiveness of the feedback derived of self-observation in the learning of motor skills (Salmoni et al., 1984; Newell, 1991; Kernodle and Carlton, 1992; Janelle et al., 1997; Silva et al., 2016). Moreover, starting with an hetero-observation to provide information on what is well done, what should be improved, and how it should improve (these last two pieces of information are known as feedforward) has proved to have a positive effect in order to modify different behaviors in different settings (Newell, 1991; Kernodle and Carlton, 1992; Buggey, 2007; Dowrick, 2012; Mason et al., 2016). As far as we know, the combination of feedback based on self-observation (the observed and the observer are the same person) and hetero-observation (the observed and the observer are different people, e.g., worker and technician) has not been incorporated in the occupational MMH training. Based on the studies reviewed, these components could be particularly beneficial to improve the training of workers with special responsibilities in fostering safety habits in workplaces (e.g., supervisors).

On the issue of observational instruments, there are few studies specifically evaluating behavioral change in manual handling, and they generally rely on observational methods developed with the aim of assessing MSD risk (e.g., Snook and Ciriello, 1991; Daltroy et al., 1993; Waters et al., 1993; Best, 1997; Lortie and Baril-Gingras, 1998; Nygård et al., 1998; Monnington et al., 2002; Chang et al., 2003; Marras and Karwowski, 2006; Batish and Singh, 2008; Village et al., 2009). However, these kinds of instruments do not allow for the assessment of behavior change in the way of characterizing body positions adopted during the process of manual handling execution, and this is crucial to create feedback on performance instead of only feedback on results (Salmoni et al., 1984; Kernodle and Carlton, 1992; Janelle et al., 1997; Magill, 2011).

The general purpose of this study was to develop and justify observational instruments that combine two uses during manual handling training on back pain prevention. On the one hand, change assessment in worker behavioral patterns. On the other hand, its use as a source of feedback based on systematic self-observation by workers and the hetero-observation of workers by a technician. The development of observational instruments was based on observational methodology (Anguera, 2003) and will be reported following the Guidelines for Reporting Evaluations Based on Observational Methodology (GREOM) (Portell et al., 2015; included in the EQUATOR library). This methodological approach allows the systematic analysis of spontaneous behavior in a natural environment using a set of dimensions and categories to evaluate behavior changes and temporal patterns using T-pattern analysis (Magnusson, 1996, 2000, 2018; Casarrubea et al., 2018), lag sequential analysis (Bakeman and Quera, 2011; Quera, 2018) or polar coordinate analysis (Sackett, 1980; Morillo et al., 2018; Rodríguez-Medina et al., 2018). Observational methodology is well established in several fields (Blanco-Villaseñor et al., 2010; Anguera and Hernández-Mendo, 2016; Arias-Pujol and Anguera, 2017; Cerezo et al., 2017; Escolano-Pérez et al., 2017; Izquierdo and Anguera, 2018; Pérez-Tejera et al., 2018; Santoyo and Mendoza, 2018). Nonetheless, their use to provide feedback as an intervention component within an occupational MMH training, as well as their use to assess behavioral change derived from training is innovative. We use the term SsObserWork (Systematic Self-Observation of Work) to label our application of the observational methodology for workplace health promotion. The SsObserWork design and evaluation can be seamlessly integrated into the research framework for the development and implementation of interventions preventing work-related musculoskeletal disorders proposed by van der Beek et al. (2017). Specifically, the aims of this paper were: (1) justify and develop an hetero-observation instrument with a level of analysis convenient to derive a parallel version for systematic self-observation during the training on MMH technique; (2) provide evidence on the inter-rater reliability of the hetero-observation instrument; (3) provide evidence on the usability of the self-observational instrument and its usefulness perceived; and (4) provide evidence on the use of the hetero-observational instrument to create feedback based on T-pattern analysis and polar coordinate analysis.

## Methods

Concerning the methodological approach, this work is enclosed within the mixed methods perspective that are characterized by the integration of qualitative and quantitative elements. This integration was carried out from the “connect” option (Creswell and Plano Clark, 2011). Systematic observation grounded in observational methodology (Anguera, 2003) was applied, because it was suitable in relation to the proposed aim. Recently it has been considered in scientific literature that observational methodology studies, in which the QUAL-QUAN-QUAL macro stages take place, can also be considered mixed method studies in certain circumstances, as they apply unconventional approaches (i.e., not based on frequency counts) to quantitize qualitative data (Sánchez-Algarra and Anguera, 2013; Anguera et al., 2017, 2018b). Researchers have been mixing qualitative and quantitative approaches from the last 20 years. Many researchers do not mix qualitative and quantitative approaches in optimal ways, but qualitative techniques can be used to enhance the development of quantitative instruments and vice versa. Its potential is very broad, and includes instrument fidelity, “maximizing the appropriateness and/or utility of the instruments used, whether quantitative or qualitative” (Onwuegbuzie et al., 2010, p. 57).

We used systematic and direct observation. According to the possible study designs, described in observational methodology (Anguera et al., 2001; Portell et al., 2015), the design used can be classified as nomothetic, follow-up and multidimensional. It is nomothetic because we conducted a parallel analysis of a group of workers. We classify as incomplete follow-up given the observation during two training sessions and the intra-sessional follow-up (intensive or continuous monitoring of events throughout observation sessions in order to obtain behavioral dynamic indicators or sequential data). The multidimensional nature of the design was determined for the multiple criteria included in the purpose-designed observation instrument. Additionally, the quantitative approach was complemented with data collected from a cross-sectional design in order to obtain evidence of usability and usefulness.

### Observation Instruments Development

The SsObserWork instruments were developed in three phases. In the first phase, a review of the scientific literature was conducted to establish the research background. We searched the Scopus, Web of Science, PubMed and Google Scholar databases for studies on the MMH training phases (Hsiang et al., 1998; Plamondon et al., 2014), and mostly studies on the effect of the back position (Anderson and Chaffin, 1986; McGill, 1997; Larivière et al., 2000; Straker, 2003b; Mörl et al., 2005; Kahrizi et al., 2007; McGill, 2007; Mawston and Boocock, 2012), feet position (Kirby et al., 1987; Authier et al., 1996; Kingma et al., 2004; Kingma et al., 2006; Demaret et al., 2007; Zhou et al., 2013), knee position (Hagen et al., 1993; Straker, 2003a,b; Kingma et al., 2004; Mörl et al., 2005), arms and load position (Hsiang et al., 1997; Marras et al., 1999; Demaret et al., 2007; Faber et al., 2009; Colombini et al., 2013) on MMH and their impact on the risk of back disorders. Based on this review and considering the objective to establish an instrument similar for hetero-observation and systematic self-observation, the granularity of the codes (Bakeman and Quera, 2011; Anguera et al., 2018a) was established. It was done considering that the procedure of modeling movements by observation with the objective of a performance description can vary from a micro to macro (or molecular to molar) level of granularity or specificity. On one side, codes can be created to capture minimum details (e.g., repeated measurement of movements during the process based on sensor-based high-resolution) while on the other end of the continuum they can be relatively broad (e.g., general assessment of balance and coordination at the end of the process). We selected a medium level of granularity for the decomposition into categories of the MMH process. With this level of granularity, the instrument for worker hetero-observation will be able to generate sequential data of their performance. Moreover, this level of granularity seems molar enough to allow us to obtain a parallel version understandable for the worker during the implementation of the systematic self-observation; we will refer to this version as self-observation instrument, hereinafter S-O instrument.

In the second phase, the observational context was established, and a pilot testing of a preliminary version of the hetero-observation (H-O) instrument was performed. The instrument was developed focusing on a standard MMH task in a training context in which attention is paid to lifting, carrying and lowering actions done spontaneously by the employee from a sagittal plane. The recording requirements were established based on a previous review (Kilbom, 1994; da Silva et al., 2014). The instrument was developed as a combination of field format and category systems, and it was created with nine dimensions. For the first eight dimensions we built category systems to codify the positions of different body segments and sub-segments: feet, feet with respect to floor, knees, back, arms with respect to legs, arms with respect to back, elbows, and shoulders. The last dimension was a catalog of codes to identify the phases. A standardized training manual was developed with definitions and diagrams for each category and code. This first version was applied in a pilot study implemented in a company in the metallurgical sector. Pilot testing of this preliminary version was performed by two observers trained in ergonomics who applied the instrument to the observation of 16 workers doing 160 MMH tasks. Data quality was evaluated by an inter-observer reliability analysis, using conventional Cohen’s Kappa agreement index. The Kappa values of the dimensions ranged from 0.39 to 0.75, with a median of 0.65, indicating moderate agreement (Landis and Koch, 1977).

In the third phase, the H-O instrument and the manual were optimized and the self-observation (S-O) version was created. Four external experts in different areas took part in this process: a physiotherapist specialized in patient transfer, two specialists in occupational medicine and safety, ergonomics, psychology and industrial hygiene, and an expert in physical activity and sports science, specifically in physical activity at work. These experts participated in qualitative interviews on the appropriateness and representativeness of the dimensions, categories and training context. The interviews were organized as an iterative process whereby each interview informs the next, and subsequent interviews are used to explore the weaknesses raised in previous interviews (Brod et al., 2009). Interviews were conducted by MP and AS-M. During this phase, the number of dimensions and categories was reduced, the definitions were improved, the classification criteria to define supra-categories (recommended or non-recommended to prevent health risk) were established, recording rules were clarified, and the observers training process was improved.

### Participants and Samples

A convenience sample was initially composed by 53 blue-collar workers of a food processing company in Catalonia (Spain), who were interested in participating in two sessions of a multicomponent training on health promotion in the workplace. The sample participants were 24 men (45%) and 29 women (55%), of which 4% were between 18 and 28 years old, 38% were between 29 and 39 years old, 30% were between 40 and 50 years old and 28% were more than 50 years old. Participants did not have chronic bone, muscle or joint pathology in the trunk, knees, nor chronic or acute pain diagnosed by a specialist. While attending training sessions, workers were video-recorded during box manipulation (see next section).

On the reliability study of the H-O instrument the sampling unit was the box-manipulation. From a total number of 530 boxes manipulated, 84 box-manipulations were randomly selected for the reliability study. The hierarchical stratification sampling scheme used took into account the position of the box within session and between sessions. This sampling scheme ensured that a minimum of 77% of the participants were represented on this box-manipulation sample.

Regarding the study on usability of the S-O instrument, the participants were a subsample of 27 workers who used the S-O instrument in two separate self-observation sessions. Participation in this study was voluntary, but the opportunity to take part in two self-observation sessions was randomly established. The participant subsample were 10 men (37%) and 17 women (63%), of which 41% were between 29 and 39 years old, 37% were between 40 and 50 years old and 22% were more than 50 years old; no statistically significant differences were observed on these variables regarding the general sample.

The Ethics Committee of the Universitat Autònoma de Barcelona approved the study protocol. In accordance with the principles of the Declaration of Helsinki, participants were informed that they were being filmed. They were shown the location of the video cameras, which were positioned discretely to minimize reactivity bias. Informed consent was also obtained.

### Instruments

According to the GREOM (Portell et al., 2015), the reporting of systematic observation studies must clarify the distinction between observation instruments (i.e., purposed-designed instruments to analyze a given participant; where development is the main objective of this paper and it will be presented as a part of the results) and recording instruments (i.e., those used to record and code data according to the dimensions established by the observational instrument). In this study, the recording instrument was LINCE (v.1.2.1) (Gabin et al., 2012; Hernández-Mendo et al., 2014). Box manipulation was registered with Sony HD video cameras and videotapes were transferred to Toshiba Portege laptops for their codification. The following computer software was used to perform the data analysis: GSEQ (v.5.1) (Bakeman and Quera, 2011), HOISAN (v.1.6.3.3) (Hernández-Mendo et al., 2012), and THEME (v.6.0 Edu) (Magnusson, 2000).

### Procedure

As a part of a multicomponent training in health promotion at the workplace, workers were video-recorded while having to lift, carry and lower 5 boxes (8 kg each). The video camera was positioned at the workers’ hip height and the plane was sagittal. All observations took place in company spaces adapted to training. After MMH performance and recording, a subsample of 27 workers observed their own performance using the S-O instrument. During this self-observation task the technician provided feedback and feedforward considering the classification associated to H-O and S-O instruments (Table 1). These workers repeated the MMH performance recording and systematic self-observation task 3 weeks later. At the end of this second systematic self-observation task, workers were required to answer five questions on usability and usefulness perceived of the S-O instrument, which were adapted from previous studies (Tsakonas and Papatheodorou, 2008; Zapata et al., 2015). The usability questions explore the understandability of the terminology, images, aesthetic appearance and layout. The usefulness questions explore the worker’s perceptions regarding the instrument’s ability to improve their knowledge on MMH technique and their behavior during MMH. A 10-point response scale was used for all questions (from 1 – very low – to 10 – very high).

**Table 1:** Dimensions and category systems of SsObserWork instruments: hetero-observation (H-O) and self-observation (S-O) version.

H-O instrument Category systems	Code	Classification^a^(Recommended -R- or Non- Recommended -NR-)	S-O instrument^b^
**Dimension 1. Feet**		
Symmetric feet behind the load	p1	NR, during LF and LW phases.	**✓**
Asymmetric feet behind the load	p2	NR, during LF and LW phases.	**✓**
Symmetric feet beside the load	p3	R, during LF and LW phases.	**✓**
One foot beside the load and the other behind it	p4	HR, during LF and LW phases.	**✓**
Walking	ppv		
**Dimension 2. Knee joints**		
Extension – slight flexion	rex		**✓**
Moderate flexion	rmo	R, during LF and LW phases. It is just considered NR when the highest position and the upright (0 cm) position are concurring.	**✓**
Maximum flexion	rsq	NR, during all MMH phases.	**✓**
Walking	rcv		
**Dimension 3. Back**		
Neutral	tne	R, during all the MMH phases.	**✓**
Flexion	tF	NR, during all the MMH phases.	**✓**
Maximum flexion	thip	NR, during all the MMH phases.	**✓**
Extension	tEx	NR, during all the MMH phases.	**✓**
**Dimension 4. Elbow joints**		
Extension – slight flexion	b1	R, during all the MMH phases.	**✓**
Flexion	b2	NR, during all the MMH phases.	**✓**
**Dimension 5. Load position**			
Close to the body	ap	R, during all the MMH phases.	**✓**
Separated from the body	se	NR, during all the MMH phases.	**✓**
**Dimension 6. Interaction between back tilt and displacement**		
Tilt at 0 cm	sin	R, during LF and LW phases.	
Tilt at > 0 cm	non	NR, during all the MMH phases.	
Upright at 0 cm	f13	R, during the highest position of LF and LW phases.	
Upright at > 0 cm	anda	R, only during CA phase.	

*^*a*^The classification as recommended (R) or non-recommended (NR) is defined in relation to the MMH code phases: LF-Lifting [with three subphases: initial position (f1a), lowest position (f1b), highest position (f1c)]; CA-Carrying (f2); LW-Lowering [with three subphases: highest position (f3b), lowest position (f3c), final position (f3d)]. ^*b*^The check mark (****✓****) indicates the presence of the category in the S-O instrument.*

For the reliability study of the H-O instrument we adapted a double approach justified by Arana et al. (2016). A first block of codes (block AB) was generated by two members (AS-M, MP) using a process of qualitative consensus agreement in the application of the H-O instrument to the 84 units. Parallel to this, a senior technician for work hazard prevention (who did not participate in the instrument development) was trained in the use of the H-O instrument for 9 h with specific materials (the box-manipulation used for this training was not included in the sample). This technician codified the 84 units, acting as an independent observer, and creating a second block of codes (block C).

### Data Analysis

#### Time-Unit Kappas and Event-Alignment Kappas

Considering the purpose of this paper, we used a demanding approach to the agreement study, emphasizing observer agreement regarding the data collected (not with scores derived from such data). For each category system, data were recorded as time-event sequential data (Bakeman and Quera, 2011), that is, mutually exclusive and exhaustive (ME&E) codes for each dimension had been assigned to events as they unfold over time (micro-coded). We applied the agreement study algorithms for time-event sequential data proposed by Bakeman et al. (2009): time-unit kappas and event-alignment kappas. Both time-unit and event-based kappas were computed using GSEQ (v.5.1) (Bakeman and Quera, 2011; Quera, 2018). Time-unit kappa examines interrater agreement in time (i.e., how long agreement and disagreement lasted). On this data we consider it acceptable not to count minor errors of timing on the order below 0.5 s and we define five levels of tolerance: 0.5, 1, 1.5, and 2 s. Event-alignment kappa examines interrater reliability on code order for timed events (i.e., onsets and durations). In this procedure, GSEQ aligns codes using a predefined algorithm (Bakeman et al., 2009) and examines agreements, omission and commission errors. We also use the mentioned five levels of tolerance and an 80% event overlap.

#### T-Pattern Detection and Analysis

T-pattern analysis was proposed and developed by Magnusson (1996, 2000). This analysis allows for detection of hidden or nonobvious temporal patterns in behavior that are not always visible. The detection algorithm first identifies significant (non-random) recurrences of any two events (in T-pattern analysis “event” refers to the configuration of codes from each dimension of the observational instrument) within a similar temporal configuration (critical interval) in real-time behavioral data and then proceeds to identify hierarchical relationships with any other antecedent or subsequent events. T-pattern analysis involves the use of an algorithm that calculates temporal distances between behaviors and analyzes the extent to which critical intervals remain invariant relative to the null hypothesis, that each behavior is independently and randomly distributed over time. The algorithm developed by Magnusson (1996, 2000) has been implemented in the THEME_TM_ software (Patternvision Ltd., Iceland). Data from this study was analyzed using Theme 6.0 Edu. Once T-patterns have been detected it is possible to use this new information in different ways (Casarrubea et al., 2015, 2018). One approach is based on the analysis of pattern sets and another approach is to analyze patterns individually (e.g., Castañer et al., 2017). This second approach is the one used on this paper. From that approach it is crucial to be transparent regarding the qualitative and quantitative filters used to select T-patterns to suit the objective of the analysis (Amatria et al., 2017). Considering the purpose of our T-pattern selection, which is to help employees analyze their own MMH in a better way, the filters used were first quantitative and secondly qualitative. The quantitative filters were: (1) minimum one occurrence of the pattern on each MMH, (2) frequency of occurrence higher than 3; (b) significance level of 0.005 (0.5% probability of critical interval being due to chance). The qualitative filters were (applied as a lexicographic decision rule): (1) maximum length (number of event-types in the terminal string of a pattern), (2) maximum level (number of hierarchical levels in a pattern), (3) different event-types (configuration of codes), (4) items (codes) related to recommendable position, (4) maximum duration. The results were validated by simulation, through data randomization on five occasions, accepting only patterns for which the probability of randomized data coinciding with real data is zero.

#### Polar Coordinate Analysis

Polar coordinate analysis, which was proposed by Sackett (1980), combines adjusted residuals from lag sequential analysis (Bakeman, 1978) and the Z_sum_ statistic (Cochran, 1954). It involves the detection of significant associations between a behavior of interest (referred in polar coordinate analysis as *focal behavior*) and other behaviors (referred as *conditional behaviors*). Polar coordinate analysis is based on the complementarity between two analytical perspectives: prospective and retrospective, concerning the focal behavior as zero point. The Z_sum_ statistic provides a representative value for a series of independent values (adjusted residuals at different prospective or retrospective lags) to produce prospective and retrospective Z_sum_ values. Prospective Z_sum_ values are represented in X axis, and Z_sum_ retrospective values in Y axis. The resulting values and their sign (positive or negative) determine the quadrant in which the different vectors are located and indicate their respective lengths and angles (Sackett, 1980). The value of angles implies a necessary adjustment of the trigonometrical value, as a function of the quadrant. Vectors provide information on the nature of the relationship (prospective/retrospective and activation/inhibition) between a focal behavior, which is equivalent to a given behavior in lag sequential analysis, and each conditional behavior that we have proposed in our study (see results section). We used the genuine retrospective approach. The concept of genuine retrospectivity (Anguera, 1997) was introduced at a later stage to improve the classic concept of retrospectivity (Sackett, 1980). This approach considers negative lags from a backwards rather than a forward perspective, i.e., it looks at what happened from lag 0 back to lag -1 rather than from lag -1 to lag 0, and the same in successive lags. Sackett (1980) recommended using the same number of prospective and retrospective lags. Based on experience to date (Sackett, 1987; Anguera et al., 2018c), five prospective lags and five retrospective lags (-5 to +5) were analyzed. The meaning of the vectors varies according to the quadrant in which they are located, and the position in one quadrant or another is determined by the combination of positive or negative signs on prospective and retrospective Z_sum_ values. In quadrant I (+ +), the focal and conditional behaviors activate each other; in quadrant II (- +), the focal behavior inhibits and is activated by the conditional behavior; in quadrant III (--), the focal and conditional behaviors inhibit each other; and in quadrant IV (+ -), the focal behavior activates and is inhibited by the conditional behavior. Vector length indicates the strength of the association between focal and conditional behaviors. The HOISAN program (v.1.6.3.3) (Hernández-Mendo et al., 2012, 2014) was used to calculate adjusted residuals, Z-values, and vector length and angles; the program includes a feature to produce results in graph form.

## Results

### SsObserWork Systematic Observation Tools: H-O Instrument and S-O Instrument

Based on literature review and expert opinion, the H-O instrument proposed was a combination of a field format and category systems (Anguera, 2003; Portell et al., 2015). It was composed of six dimensions (feet, knee joints, back, elbow joints, load position, and interaction between back tilt and displacement) and twenty-one categories (Table 1). Additionally, these six dimensions had a formal category null (empty set) which marks off an unobservable action. The set of categories corresponding to each dimension met the requirements of ME&E. Additionally, there was a structural dimension which defined MMH phases. In Table 1, dimensions and categories are shown (details in Supplementary Table S1). Based on the scientific review described in the previous section, the twenty-one categories were classified according to their effect on health. On the third column of Table 1, the derived classification as a recommended or non-recommended position to be adopted during MMH is summarized. This data was the base to create the modified version of the H-O instrument to construct the S-O instrument and to create feedback. The last column of Table 1 indicates the categories included on the S-O instrument with a check mark. The S-O instrument kept all dimensions except the most complex one (interaction between tilt and displacement) and all categories were defined graphically.

**FIGURE 1:**
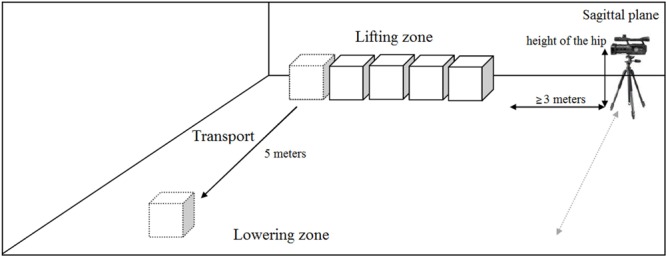
Space disposition for MMH in formative context.

**FIGURE 2:**
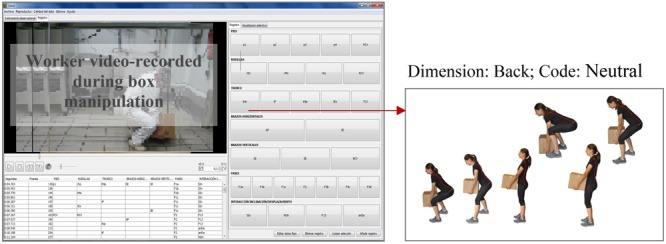
Sample interface used to codify. Left side shows the recording instrument. Right side shows the visual part of one category definition in the H-O instrument. Written informed consent was obtained from the depicted individual for the publication of these images.

Both instruments can be used to observe the MMH task in formative training addressed to improve MMH in diverse work environments at a range of industrial sites. The application requirements were: (1) a conventional video camera positioned at the workers’ hip height; and (2) the instrument to record observational data. Figure 1 shows space disposition for MMH in formative context and Figure 2 shows the interface used to codify. The observational instruments, the record instrument to automatize codification and the training program could be facilitated upon request.

Average time required to apply the observation instruments were (time by box in the generic setting illustrated in Figure 1): 9 s for MMH recording, 6 min for the codification of the H-O instrument by the technician, and 3 min for the codification of the S-O instrument by the worker.

### Inter-Rater Reliability Testing of the H-O Instrument

The values of agreement statistics across observers (AB register vs. C) for each dimension are summarized in Table 2. Supplementary Tables S2, S3 list the values of agreement statistics for each category. In addition to kappa values, Table 2 includes the kappa maximum and percentage of agreement.

**Table 2:** Agreement between register AB and C for each dimension, presenting kappa based on events and kappa based on time units for each tolerance level, as well as percentage of agreement and kappa maximum.

		Based on events			Based on time units		
Dimension	Tolerance level	% agreement	Kappa	Kappa max.	% agreement	Kappa	Kappa max.
Feet	0.5	92	0.87	0.93	97	0.92	0.95
	1.0	93	0.89	0.94	97	0.93	0.95
	1.5	94	0.90	0.94	97	0.93	0.95
	2.0	94	0.90	0.94	97	0.93	0.96
Knee joints	0.5	89	0.86	0.97	99	0.97	0.98
	1.0	90	0.87	0.97	99	0.98	0.99
	1.5	91	0.87	0.97	99	0.98	0.99
	2.0	91	0.87	0.97	99	0.99	0.99
Back	0.5	89	0.83	0.94	94	0.90	0.97
	1.0	92	0.88	0.95	96	0.93	0.97
	1.5	91	0.87	0.95	97	0.94	0.97
	2.0	92	0.88	0.95	97	0.94	0.97
Elbow joints	0.5	86	0.72	0.88	96	0.92	0.94
	1.0	89	0.78	0.89	96	0.93	0.94
	1.5	91	0.82	0.89	97	0.93	0.94
	2.0	91	0.82	0.89	97	0.93	0.94
Load position	0.5	87	0.76	0.95	95	0.91	0.94
	1.0	91	0.81	0.94	97	0.94	0.96
	1.5	93	0.85	0.93	98	0.97	0.98
	2.0	93	0.85	0.91	99	0.98	1
Interaction between back tilt and displacement	0.5	84	0.78	0.92	97	0.96	0.98
	1.0	86	0.80	0.92	98	0.97	0.98
	1.5	87	0.82	0.92	98	0.97	0.99
	2.0	87	0.82	0.92	99	0.98	0.99

Regarding the results obtained from event-based kappas for each dimension, the kappa values ranged from 0.72 (for elbow joints criterion at 0.5 tolerance level) to 0.9 (for feet criterion at 1.5 tolerance level), indicating good to very good agreement according to the criteria of Landis and Koch (1977) and Altman (1991). Regarding event-based kappas for the categories (Supplementary Table S2) and considering the higher demanding tolerance level (0.5 s), the kappa values ranged from 0.5 (for the “symmetric feet beside the load” category included in the feet criterion) to 0.97 (for the “walking” category included in the feet criterion), indicating moderate to very good agreement according to the criteria of Landis and Koch (1977) and Altman (1991). Values indicating moderate agreement happened in one of the 21 categories included in the H-O instrument; thus, 95% of the categories presented kappa values indicating good to very good agreement.

Regarding the results obtained from time-unit kappas for each criterion, the kappa values ranged from 0.90 (for the back criterion at 0.5 tolerance level) to 0.99 (for the knee joints criterion at 2 tolerance level), indicating very good agreement according to the criteria of Landis and Koch (1977) and Altman (1991). Regarding time-unit kappas for the categories (Supplementary Table S3) and considering the higher demanding tolerance level (0.5 s), the kappa values ranged from 0.53 (for the “symmetric feet beside the load” category included in the criterion feet) to 0.99 (for the “walking” category included in the criterion feet), indicating moderate to very good agreement according to the criteria of Landis and Koch (1977) and Altman (1991). The values indicating moderate agreement happened in just one of the 21 categories included in the H-O instrument; thus, 95% of the categories presented kappa values indicating good to very good agreement.

### Usability and Usefulness Perceived of the S-O Instrument

Table 3 reports the median, mean and standard deviation of the usability and usefulness perceived by workers who apply the S-O instrument during two separate sessions. The last column shows the results of the one-tailed sign test using 7.5 as a cut point. For all the studied aspects, the median was statistically greater than 7.5 (on a scale of 1–10, being 10 the best).

**Table 3:** Usability and usefulness perceived of the S-O instrument.

Item^a^	*Md*	*M*	*SD*	Sign test *p^b^*
Usability: understandability of the terminology	8	8.44	1.50	<0.0001
Usability: understandability of the images	9	8.81	1.52	<0.0001
Usability: aesthetic appearance and easy and clear layout	9	8.78	1.25	<0.0001
Usefulness: behavior improvement during the MMH	9	8.81	1.42	<0.0001
Usefulness: knowledge improvement on MMH technique	10	9.11	1.19	<0.0001

*Descriptive statistics and one-tailed sign test results. *^*a*^10-point response scale was used for all questions (from 1 – very low – to 10 – very high). ^*b*^One-tailed sign test using 7.5 as a cut point to define dichotomy.**

### Use of the H-O Instrument to Create Feedback Based on T-Pattern Analysis and Polar Coordinate Analysis

**FIGURE 3:**
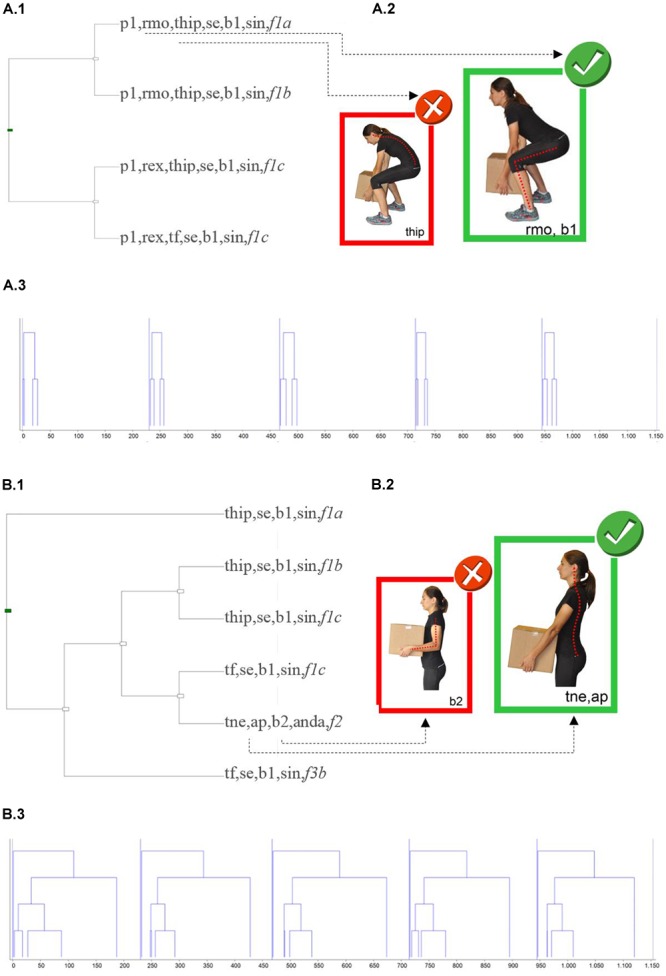
Schematic representation of two T-patterns **(A,B)**, that occur throughout the MMH process of the worker WO1. **(A.1,B.1)** Include the pattern tree graph. **(A.2,B.2)** Include pictures highlighting details of the configurations (the recommended positions in larger sizes than those non-recommended); for confidentiality reasons, the workers’ images have been replaced by pictures from the H-O instrument manual. **(A.3,B.3)** Includes the instance graph. Written informed consent was obtained from the depicted individual for the publication of these images.

**FIGURE 4:**
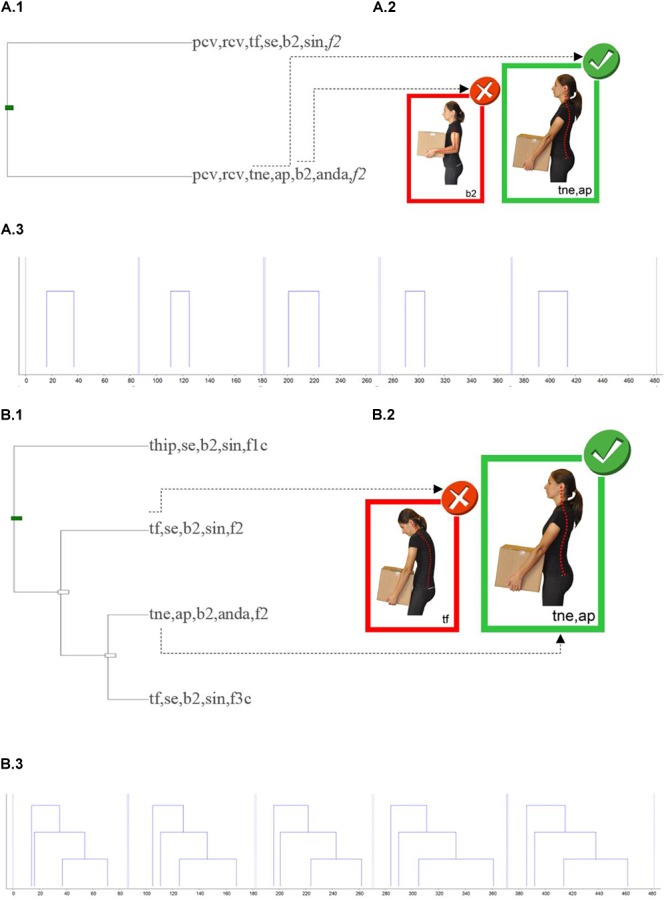
Schematic representation of two T-patterns, that occur throughout the MMH process of the worker WO2. **(A.1,B.1)** Includes the pattern tree graph. **(A.2,B.2)** Includes pictures highlighting details of the configurations (the recommended positions in larger sizes than those non-recommended); for confidentiality reasons, the workers’ images have been replaced by pictures from the H-O instrument manual. **(A.3,B.3)** Includes the instance graph. Written informed consent was obtained from the depicted individual for the publication of these images.

**FIGURE 5:**
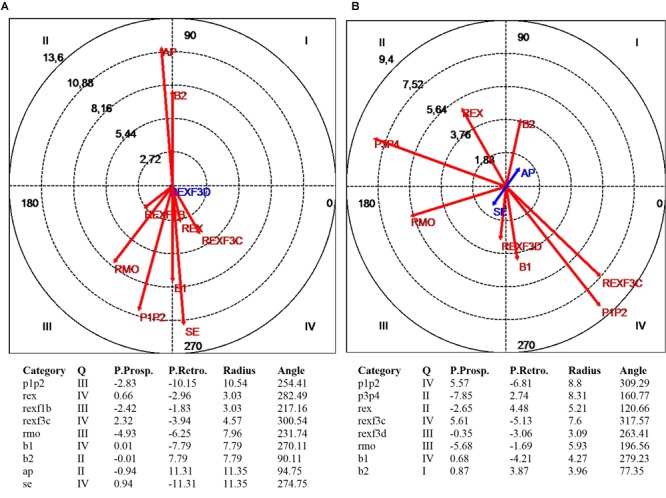
Maps of polar coordinate analysis for worker WO1 **(A)** and worker WO2 **(B)**. Under each graph the table with the polar coordinate analysis results is presented for the statistically significant associations. The focal category is the neutral back position (tne). The conditional behaviors are: non-recommended feet position (p1p2), recommended feet position (p3p4), knees flexed moderately during lifting and lowering (rmo), knees extended or slightly flexed during lifting and lowering (rex), knee joints in a non-recommended position in initial/lifting-lowest/lowering-lowest/final position (rex1a/rex1b/rex3c/rex3d), knees are flexed severely (rcv), elbow joint is extended or slightly flexed (b1), one or both elbow joints are flexed (b2), load close to the body (ap), load separated to the body (se).

To illustrate the functionality of the H-O instrument to create feedback on performance, two workers were selected (we refer to them as WO1 and WO2), both males, aged between 40 and 50 years old, who have equivalent values in terms of recommended position when they are examined globally throughout the session (global values between P40 = 53.3% and P50 = 54.8% of the distribution of relative durations). Using the GREOM terms (Portell et al., 2015; Chacón-Moscoso et al., 2018), duration is a static behavioral indicator, but the granularity of the H-O instrument is be able to define additional and more complex dynamic indicators. Based on these behavioral dynamic indicators, joined with the possibilities of sequential analysis techniques, Figure 3–5 illustrate the new information that can emerge from the observational record.

For each worker, Figure 3, 4 show the two most relevant T-patterns detected by applying the selection criteria described in the method section. For each T-pattern two diagrams are presented. Firstly, the tree graph pattern (Figure 3A.1,B.1, 4A.1,B.1) shows which event types (configurations of concurrent codes) are included in the pattern and how they are connected. Secondly, the instance graph (Figure 3A.3,B.3, 4A.3,B.3) provides information about the real-time pattern structure (the time period represented only includes the observation periods in which the worker loads, displaces, or unloads each load). On the right side of the tree graph pattern, the pictures highlight configuration details (for confidentiality reasons the workers’ images have been replaced by photographs from the H-O instrument).

Figure 5 summarizes the results of polar coordinate analysis for each worker. Each graph represents the statistically significant associations (activation or inhibition) between the focal and conditional behaviors. The focal behavior on this analysis was “neutral back position.” The conditional behaviors were the categories of the dimensions feet, knee joints, elbow joints and load position, after applying two kinds of code transformations based on the classification criteria included in Table 1. The first was a merger of categories of the feet dimension and the second the creation of new superordinate codes combining the knee joints and phase dimensions (the new codes have been created by joining the codes of the original categories that were already defined in Table 1). The association is shown both quantitatively (vector length) and qualitatively (quadrant I, II, III, or IV). Under each graph, the table with the polar coordinate analysis results is presented for the statistically significant associations.

The qualitative information which can be deduced from these analyses is presented below to illustrate how they provide useful resources to improve the informative quality of the feedback and feedforward on worker performance.

Regarding worker WO1, the first T-pattern analysis was applied at 693 micro-coded observational data which were structured in 99 configurations (33 different event-types). Taking into account all the dimensions of the H-O instrument, the most complete pattern identified for 100% of the MMH performed only showed a regular structure in event-types related to the lifting phase (Figure 3A.1,A.3). This pattern also showed that knee joints, elbow joints and interaction are dimensions that remain in a recommended position during this regular pattern of behavior. Theme also allows more detailed analysis of a part of the dimensions. In this case, position analysis related to upper body dimensions was applied (Figure 3B.1,B.3). The most complex pattern obtained for this subset of configurations, which is significant for 100% of the MMH performed, connects the way of performing the lifting with load displacement, with lowering performance not being structured enough for it to be incorporated into the pattern. It is also worth noting the regularity of the co-occurrence between the neutral back position and the position close to the body detected by Theme (see the biggest picture in the Figure 3B.2).

Polar coordinate analysis showed that the neutral back position of the worker WO1 (Figure 5A) was sequentially associated with the dimensions: feet, knee joints, elbow joints and load position. From this analysis, the most interesting relationship that can be presented as feedback to worker WO1 was about the feet (code P1P2) in quadrant III, where the vector represents a mutually inhibitory sequential relationship between a neutral back position and a non-recommended position of the feet during lifting and lowering phases. In quadrants II and IV two other strong relationships can be seen, both of which are of asymmetric dependence. The first relationship pointed out is that the recommended position of the back inhibited load position close to the body, although load position close to the body activated the neutral back position. The relationship shown in quadrant IV is the mirror image: the recommended position of the back activated the load position separated from the body and this last inhibited the recommended position of the back.

Regarding worker WO2, the first T-pattern analysis was applied at 525 micro-coded observational data which was structured in 75 configurations (30 different event-types). Taking into consideration all the dimensions of H-O instrument, only one common pattern was detected in 100% of the MMH performed. This very simple pattern structures behavior during the carrying phase (Figure 4A.1,A.3). This pattern detects regular associations between an event-type and especially non-recommended co-occurrences (starting displacement with the back tilted due to an anterior hip flexion, in the final position of the lowering phase and with the rest of the dimension in a non-recommended position) and another event-type which includes two very recommended co-occurrences (load position close to the body and neutral back position). Figure 4B.1,B.2 show the results focusing on the search of T-patterns among the subset of dimensions back, elbow joints, load position and interaction. The most complex T-pattern detected in 100% of the MMH performed, structures the temporary association previously detected (Figure 4A.1) with other configurations, all of which are not recommended, during the loading and lowering phases.

When the polar coordinate analysis was applied to worker WO2 data (Figure 5B), it was observed that the neutral back position adopted by this worker is sequentially associated with other dimensions such as feet, knee joints and elbow joints. The strongest sequential relationships were observed in quadrant IV and this indicates the following asymmetric dependence relationship: the recommended back position activates non-recommended feet and knee joint positions during the middle part of the lowering, and non-recommended feet and knee joint positions inhibit the recommended position of the back. The previous relationship is not particularly useful to use as an informative feedback for the worker. From this analysis, the most interesting relationship that can be presented as feedback for worker WO2 is about the mutually inhibitory sequential relationship (quadrant III) between a recommended back position and a non-recommended knee joint position at the end of the lowering phase (the knees were bent less than what should be recommended).

## Discussion

Our study shows the design and implementation of two systematic observation instruments of worker behavior during MMH training for back-pain prevention in the workplace. These observation instruments address a double purpose: the behavioral change assessment and the creation of feedback under a training component that we have labeled SsObserWork. Following the research framework for the development and implementation of interventions preventing work related MSD established by van der Beek et al. (2017), we assume that the etiology of back pain is multifactorial meaning that the SsObserWork approach is suggested as a piece for use in multicomponent occupational intervention, which in turn should be designed according to the “risk control hierarchy model.” We organized the discussion around two aspects. On the one hand, the novelties associated to SsObserWork instruments, and on the other, the evidence of their reliability, usefulness and usability.

The H-O instrument has been justified and developed for observing worker behavior during the MMH training process. The H-O instrument is composed of the dimensions feet, knee joints, back, elbow joints, load position, and interaction between back tilt and displacement. For each dimension an independent category system has been created; additionally, the H-O instrument includes a structural dimension related to the MMH phase. This instrument has been developed in such a way that it allows us to derive the simplified S-O instrument, whereby workers can apply self-observation during MMH. Both instruments are highly structured, and they were developed for video observation, as well as for the generation of feedback. Literature review provided the theoretical models to underpin the link between dimensions, categories and classification criteria to provide feedback. The expert judgment also provided support on the appropriateness of the dimensions and categories for the objectives established as well as their representativeness regarding the MMH training task selected.

The reliability results obtained by the H-O instrument were highly satisfactory. We have presented a thorough assessment of the agreement between the codes assigned by two research team observers and the codes assigned by a technician in occupational health and safety, previously trained in the use of H-O (Denis et al., 2002). The levels of agreement obtained provide strong evidence on the reliability of the H-O instrument. The small changes observed between kappa values when the analysis is based on time or event units are explained by the overestimation and underestimation associated with the respective algorithms used for obtaining these results, hence the recommendation has been followed to present them jointly (Bakeman et al., 2009). The results support the use of H-O (by a technician in occupational health and safety) as a reliable instrument to evaluate changes in worker behavior during an MMH training.

The use of observational techniques in ergonomics is not new at all (David, 2005). The novelty of the procedures being proposed here lies in the function of observation, in the granularity level used by the instruments, and in the connection between the instruments and the provision of feedback for the worker.

The H-O instrument has not been created for the assessment of exposure to MSD risk factors associated with a task or a workplace, it has been created for the assessment of changes in worker behavior during MMH in a formative context. This characteristic that we could call worker-centered evaluation, singularizes the H-O instrument regarding many of the observational techniques used in ergonomics (David, 2005; Takala et al., 2010).

Another remarkable characteristic of the H-O instrument arises when evaluating the effectiveness of different behavior-change-intervention approaches. Behavioral training evaluations using assessment instruments exist which focus on distal outcomes such as back injuries or wage-loss claims due to back injuries (Clemes et al., 2010). In other cases, the assessment instruments are focused on intermediate outcomes measured through self-report, such as knowledge about safe lifting and posture (Haslam et al., 2007). Back injuries are a consequence of the behavior that the training hopes to change, but it is an outcome further down the causal chain of a behavioral intervention. The assessment of a behavioral intervention should include behavioral outcomes (Michie and Johnston, 2012). The H-O instrument can remedy the detected lack of instruments addressed to the assessment of the proximal effect of manual handling training on intermediate variables that link training to distal changes in employee behavior (Hogan et al., 2014; Kay et al., 2014). Moreover, the H-O instrument can contribute to the understanding of the processes in training implementation.

There are some instruments focused on the evaluation of changes in worker behavior using video observation (Gattinger et al., 2014), but another important peculiar feature of the H-O instrument is its level of granularity. The H-O medium level of granularity requires video observations, because this allows a large number of items to be assessed, as video films can be replayed several times in order to observe the dimensions separately. A more decreased granularity could require the use of monitoring instruments such as sensors that are attached directly to the worker for the measurement of exposure variables at work. The use of these direct measurement systems can provide large quantities of highly accurate data, however the feasibility of this approach in the workplace has been highly controversial (David, 2005; Mgbemena et al., 2017). We have selected a medium level of granularity, which has been used for the modeling of complex behavior in the work setting (Neumuth et al., 2010), but as far as we know it has not been applied for training in the field of occupational risk prevention and health promotion in the workplace. The rationale for this medium level of granularity lies, firstly, in the purpose of the instrument which is the assessment of behavior change during the training intervention process, instead of the MSD risk assessment. Secondly, in the interest to enable the feasibility of the H-O assessment in different workplaces by occupational health professionals. Thirdly, in the interest to use a level of granularity that may also be used for the S-O instrument without hindering the worker’s understandability.

The usability and usefulness perceived of the S-O instrument has been highly valued by workers. Among the various concepts under investigation on usefulness and usability (Tsakonas and Papatheodorou, 2008; Akin et al., 2013; Zapata et al., 2015), our questions explored understandability, aesthetic appearance and layout, worker perception regarding the instrument’s ability to improve their knowledge and their behavior during MMH. Workers reported very favorable assessments toward all the usability and usefulness attributes explored and it can be interpreted as support for the selected level of granularity.

In addition to being convenient for the S-O instrument, the granularity selected allows the H-O instrument to generate suitable data to make sequential analysis able to uncover “hidden time patterns” (Magnusson, 2000) in the behavior during the training process. This paper shows how it can be used to improve the informative quality of the feedback and feedforward on worker performance. To illustrate the usefulness of the instrument for this purpose, we present the data of two workers who would receive very similar feedback if only summary indicators of the global results of their MMH are taken into account (e.g., the global proportion of time with a recommended position of the back). The results show the wealth of information that can be extracted to inform workers of different regular aspects of their execution. The feedback/feedforward build from this information combined with the self-observation made by the worker himself or herself is the base of the SsObserWork intervention component. This approach is in line with previous studies which established the importance of different forms of feedback used in combination with video feedback (Wulf et al., 2010; Ste-Marie et al., 2013; Lim et al., 2015). Systems already exist for generating feedback that informs on awkward postures adopted by workers in real time, as well as to evaluate the ergonomic risk (Mgbemena et al., 2017). However, SsObserWork is presented here as a different approach (although it would not be incompatible). Here the feedback is raised as a training component that gives the worker material to engage in systematic self-observation and reflection on his or her non-recommended position within his or her “work gesture.” Results show how the combined use of the H-O instrument with sequential analysis techniques allows feedback to be completed based on static indicators (e.g., relative duration of trunk in non-recommendable position) with dynamic indications that describe the interaction of the worker with the load. Using the complementary methods of T-pattern analysis and polar coordinate analysis (Anguera et al., 2017) it is possible to detect how co-occurrences and recommended position sequences interact within gestures that globally can be deficient, thereby contributing toward the creation of positive feedback and feedforward (Ste-Marie et al., 2011). The contribution of these analyses can overcome the limitations of the positive self-review modeling (Dowrick, 1999) and it allows progress to be made in the delivery of feedforward that helps the worker construct a previously unachieved but possible future pattern of movements. This sort of individualized feedback can open new possibilities especially addressed to influential workers because of their basic structure position (e.g., supervisors) as well as in parallel structures (e.g., members of the health and safety committees, or older employees participating as mentors in intergenerational learning programs).

This study has both strengths and limitations. The main strength of this study is to provide two new connected instruments for systematic observation that make possible the assessment and implementation of new components to enrich interventions in the prevention of back pain associated with material handling tasks. The mixed method design based on systematic observation (QUAL-QUAN-QUAL phases) makes it possible to tackle the methodological complexity of the development, assessment and integration of the results provided by these instruments. We want to highlight the demanding reliability study presented and its very satisfactory results; this point seems remarkable considering the lack of reliability data in several assessment instruments noted by different authors (Village et al., 2009; Kadikon and Rahman, 2016).

Regarding the limitations and future directions of the research we will mention three points. Firstly, the number of companies and participants has been low. In the pilot study, a company from the metallurgical sector participated and the final version was applied in a food processing company. An area in which we consider that the SsObserWork approach could be of great interest is in training for the mobilization of patients in hospitals. The structure of the H-O instrument (combined field format and category systems) facilitates adaptations and extensions to include interaction dimensions that are essential to address the complexity of manual handling in healthcare settings (Kay et al., 2014). A second limitation has to do with the high time and material cost of the coding and sequential analysis process. We hope that future studies will advance in the automation of procedures since this will facilitate the application to more companies, as well as their integration into multicomponent training interventions. Thirdly, the results on usability and utility are only based on self-reported data from a small sample. It would be convenient to replicate this analysis with larger samples and complement what is presented here with qualitative techniques (e.g., in-depth interviews) in order to gain a deeper understanding of worker perception as well as technician perceptions on the application of these instruments.

## Conclusion

In conclusion, this work introduces the SsObserWork approach and it focuses on the development, implementation and evaluation of new instruments included in SsObserWork. This detailed study has been extremely useful for the next phase of this project consisting in the evaluation of the effect of the new SsObserwork component using a randomized parallel group trial. The results presented in this work provide empirical evidence in favor of the appropriateness and reliability of these instruments. As far as we know, the approach we propose is novel in the field of MDS disorder prevention from MMH training at the workplace. A number of convergent research areas support the use of participatory approaches to health training which allow individuals to control features of their own learning environment enhancing motor learning as well as the transfer of training to different settings (Burke et al., 2006; Ste-Marie et al., 2013; Portell et al., 2014). The combined use of the S-O instrument with external feedback based on data provided by the H-O instrument opens new possibilities for these participatory approaches, always thinking on the SsObserWork approach as a piece to integrate in multicomponent occupational intervention.

## Data Availability

The datasets for this manuscript are not publicly available for confidentiality reasons. Requests to access the datasets should be directed to mariona.portell@uab.cat.

## Ethics Statement

The Ethics Committee of the Universitat Autònoma de Barcelona approved the study protocol (Reference Number 1742). In accordance with the principles of the Declaration of Helsinki, participants were informed that they were being filmed. All participants signed the informed consent form.

## Author Contributions

MP conceived and supervised the development of the project, drafted the manuscript and she also contributed to the design of the instruments, the data collection, the data codification, and the data analysis. AS-M implemented the self-observation intervention, collected the data, codified the data, and she also contributed to the development of the project, the design of the instruments, and the drafting of the manuscript. MTA contributed to the development of the project, the data analysis, and the drafting of the manuscript. GKJ and JLL contributed to the data analysis and the drafting of the manuscript. All authors have made a substantial, direct and intellectual contributions to the work, and approved it for publication.

## Conflict of Interest Statement

The authors declare that the research was conducted in the absence of any commercial or financial relationships that could be construed as a potential conflict of interest. The reviewer AH-M and handling Editor declared their shared affiliation.
