# Supplementary material for: Support System for the Assessment and Intervention During the Manual Material Handling Training at the Workplace: Contributions From the Systematic Observation
Source: Front Psychol. 2019 Jun 5;10:1247. doi: 10.3389/fpsyg.2019.01247 (PMC6560057; doi:10.3389/fpsyg.2019.01247)
Supplement: Supplementary file 1 [file Data_Sheet_1.pdf]

Supplementary Material

Supplementary Table S1. Dimensions and category systems of the H-O instrument (Hetero-Observational version). These six dimensions have a formal category null (empty set) which is applied when the dimension cannot be observed. At the bottom of the Table (in italics) there are the structural dimensions which define MMH phases.

**Dimension** / **Category**	**Code**	**Description**	**Classification**
**Feet**			
Symmetric feet behind the load.	p1	Feet are placed symmetrically behind the load.	Non-Recommended (NR) during lifting and lowering phases.
Asymmetric feet behind the load	p2	Feet are placed asymmetrically (one more advanced than the other) behind the load.	NR, during lifting and lowering phases.
Symmetric feet beside the load	p3	Feet are placed symmetrically beside the load.	Recommended (R), during lifting and lowering phases.
One foot beside the load and the other behind it	p4	One foot placed beside the load and the other is almost behind the load (toes can be placed beside).	HR, during lifting and lowering phases.
Walking	pcv	One foot or both feet are in movement.	
**Knee joints**			
Extension - Slight Flexion	rex	Knees are completely extended or flexed slightly (≤25 degrees).	R, just in the highest position of the lifting and lowering phases.
Moderate Flexion	rmo	Knees are flexed moderately, from a slight flexion (25 degrees) to the thigh and the leg forming a right angle (90 degrees).	R, during lifting and lowering phases. It is just considered NR when the highest position and the upright (0cm) position are concurring.

	Maximum Flexion	rsq	Knees are flexed severally, more than 90 degrees of flexion (the thigh and the leg tend to get closed).	NR, during all MMH phases.
	Walking	rcv	Movement of knees as a result of foot movement and also for the action of walking.	
**Back**				
	Neutral	tne	Back maintains the three natural curvatures: cervical lordosis, dorsal kyphosis and lumbar lordosis, at any range of hip flexion. This category includes the situation in which lumbar curvature can lose its lordotic posture and adopt a neutral posture as a result of the subject's morphology.	R, during all the MMH phases.
	Flexion	tF	Back loses some natural curvatures at any range of hip flexion.	NR, during all the MMH phases.
	Maximum Flexion	thip	Lumbar adopts kyphosis position, regardless of cervical and dorsal position.	NR, during all the MMH phases.
	Extension	tEx	Back is tilted backwards from an upright posture, generating an increase of lumbar lordosis.	NR, during all the MMH phases.
**Elbow joints**				

Extension - Slight flexion	b1	Elbow joint is extended or slightly flexed (≤45 degrees approximately).	R, during all the MMH phases.
Flexion	b2	One or both elbows are flexed (approximately more than 45 degrees) until maximum flexion.	NR, during all the MMH phases.
**Load position**			
Close to the body	ap	The load is placed between the feet (at the moment of lifting and lowering), between the knees (during the course of lifting and lowering), and in contact with the hip and high thighs (during the carrying).	R, during all the MMH phases.
Separated from the body	se	The load is placed in front of the feet (at the moment of lifting and lowering), in front of the knees (during the course of lifting and lowering), and is separated of the hip and high thighs (during the carrying).	NR, during all the MMH phases.
**Interaction between back tilt and displacement**			
Tilt at 0cm	sin	Back is tilted forward while one or both feet are placed at the zone	R, during lifting and lowering phases.

		where the MMH is carrying out.	
Tilt at >0cm	non	Back is tilted forward while there is a displacement (carrying phase) which is produced at the MMH space.	NR, during all the MMH phases.
Upright at 0cm	f13	Back is upright while one or both feet are placed at the zone where the MMH is carrying out.	R, during the highest position of lifting and lowering phases.
Upright at >0cm	anda	Back is upright while there is a displacement (carrying phase) which is produced at the MMH zone.	R, only during carrying phase.
MMH phases			
Lifting			
The initial position	*f1a*	*The moment that hands grip the load.*	
The lowest position	*f1b*	*From the moment in which the load stops touching the floor, until the half part of the load surpasses knees height.*	
The highest position	*f1c*	*From the moment in which the half part of the load surpasses knees height, until the worker adopts the upright position or starts the carrying phases.*	
Carrying	*f2*	*It starts when the worker carries the load (one or both feet move their position)*	

		until both feet are placed at the lowering zone.
Lowering		
The highest position	*f3b*	*From upright position or the end of carrying phases, until the half part of the load is at knees height.*
The lowest position	*f3c*	*From the moment in which the half part of the load is under knees height, until the moment before in which the load comes into contact with the floor.*
The final position	*f3d*	*The moment that the load comes into contact with the floor.*

Supplementary Table S2. Agreement between register AB and C for each category, presenting kappa based on events for each tolerance level.

**Dimension** **Category**	**Tolerance level** 0.5s	1s	1.5s	2s
Feet				
Symmetric feet behind the load	.90	.91	.92	.92
Asymmetric feet behind the load	.79	.81	.81	.81
Symmetric feet beside the load	.50	.50	.50	.50
One foot beside the load and the other behind it	.75	.75	.75	.75
Walking	.97	.98	.98	.98
Knee joints				
Extension - Slight Flexion	.86	.87	.87	.87
Moderate Flexion	.94	.91	.92	.92
Maximum Flexion	.91	.94	.95	.95
Walking	.96	.96	.97	.97
Back				
Neutral	.90	.92	.93	.93
Flexion	.90	.91	.89	.89
Maximum Flexion	.91	.93	.94	.94
Extension	.66	.66	.66	.66
Elbow joints				
Extension - Slight flexion	.73	.79	.84	.84
Flexion	.92	.96	.96	.96
Load position				
Close to the body	.82	.85	.87	.87
Separated from the body	.88	.93	.96	.95
Interaction between back tilt and displacement				
Tilt at 0cm	.93	.95	.97	.97
Tilt at >0cm	.63	.68	.69	.68
Upright at 0cm	.81	.80	.82	.82
Upright at >0cm	.93	.95	.96	.96

Supplementary Table S3. Agreement between register AB and C for each dimension, presenting kappa based on time units for each tolerance level.

**Dimension**	**Tolerance level**			
**Category**	0.5s	1s	1.5s	2s
Feet				
Symmetric feet behind the load	.96	.96	.96	.96
Asymmetric feet behind the load	.73	.75	.76	.77
Symmetric feet beside the load	.53	.58	.58	.58
One foot beside the load and the other behind it	.77	.77	.77	.77
Walking	.97	.97	.97	.97
Knee joints				
Extension - Slight Flexion	.95	.97	.98	.98
Moderate Flexion	.96	.97	.97	.98
Maximum Flexion	.97	.98	.98	.98
Walking	.99	.99	.99	.99
Back				
Neutral	.91	.94	.95	.96
Flexion	.87	.92	.93	.94
Maximum Flexion	.96	.97	.97	.97
Extension	.64	.64	.64	.64
Elbow joints				
Extension - Slight flexion	.93	.93	.93	.93
Flexion	.93	.94	.94	.94
Load position				
Close to the body	.91	.94	.97	.98
Separated from the body	.91	.94	.97	.98
Interaction between back tilt and displacement				
Tilt at 0cm	.99	.99	.99	.99
Tilt at >0cm	.81	.84	.84	.86
Upright at 0cm	.93	.94	.95	.96
Upright at >0cm	.96	.97	.97	.98
